# LncRNA SNHG3 sponges miR‐577 to up‐regulate SMURF1 expression in prostate cancer

**DOI:** 10.1002/cam4.2992

**Published:** 2020-04-05

**Authors:** Teng Li, Yi Xing, Fan Yang, Yangyang Sun, Shaojin Zhang, Qingwei Wang, Weixing Zhang

**Affiliations:** ^1^ Department of Urology The First Affiliated Hospital of Zhengzhou University Zhengzhou China; ^2^ Department of Opthalmology The First Affiliated Hospital of Zhengzhou University Zhengzhou China

**Keywords:** miR‐577, prostate cancer, SMURF1, SNHG3

## Abstract

Prostate cancer remains one of the most prevalent cancers and the main causes of cancer‐related deaths in males. Various articles introduced that long noncoding RNAs (lncRNAs) are found in vital functions in the development and progression of cancers. Although SNHG3 (small nucleolar RNA host gene 3) has been investigated in many cancers, now researches on the role and mechanism of SNHG3 in prostate cancer are lacked. In this work, SNHG3 exerted high expression in prostate cancer cell lines. Suppression of SNHG3 inhibited cell proliferation, migration, EMT (epithelial‐mesenchymal transition) process and promoted cell apoptosis. Additionally, it was found that SNHG3 could bind with miR‐577. Subsequently, SMURF1 (Smad ubiquitination regulatory factor 1) was identified as a downstream target of miR‐577 and had a negative correlation with miR‐577. SNHG3 was found to positively regulate SMURF1 expression. Furthermore, rescue assays demonstrated that co‐transfection of pcDNA3.1/SMURF1 reversed the effects of SNHG3 knockdown in cell proliferation, migration, EMT process and cell apoptosis. SNHG3 also promoted tumorigenesis in vivo. All the results above explained that SNHG3 accelerated prostate cancer progression by sponging miR‐577 to up‐regulate SMURF1 expression, suggesting that SNHG3 may act as a biomarker for prostate cancer patients.

## Graphical abstract

Inhibited SNHG3 could suppress the progression of prostate cancer cells. MiR‐577 could bind to SNHG3 and regulate the progression of prostate cancer. SNHG3/miR‐577/SMURF1 axis was found in prostate cancer cells. SNHG3/miR‐577/SMURF1 axis could modulate the progression of prostate cancer cells. SNHG3 inhibition could reduce the progression of prostate tumor.

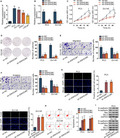

## 1 INTRODUCTION

Long noncoding RNAs (lncRNAs) are longer than 200nt and lack the potential of coding proteins. In recent years, plenty of lncRNAs have been scrutinized and the roles of them in cancer have been more specifically studied.^1^, ^2^ For example, lncRNA GHET1 can play the function of activating the cell growth of gastric carcinoma via regulating messenger RNA (mRNA) c‐Myc.^3^ Also, lncRNA SPRY4‐IT1 has been reported to enhance the cell growth, invasion and EMT progress of esophageal squamous cell cancer.^4^ Meanwhile, it has been studied that lncRNA ZFAS1 can regulate the expression of miR‐135a and modulate the cell growth of nasopharyngeal cancer.^5^ LncRNA URHC can modulate the cell growth and apoptosis by modulating the pathway of ERK/MAPK to regulate the progression of hepatocellular carcinoma.^6^ Considering the important role of lncRNA in cancers, here we are going to detect the function of lncRNA in prostate cancer.

Prostate cancer has been studied as a very frequent cancer among men and has been thought as a very dangerous cancer in America which causes significant deaths.^7^, ^8^, ^9^ It has been studied that androgen ablation and surgical treatment are the main therapies for prostate cancer.^10^ However, the 5‐year survival rate was not optimistic and further study on prostate cancer should be implemented. Many lncRNAs are dysregulated in prostate cancer.^11^ For instance, lncRNA625 is up‐regulated in prostate cancer cells and sponges miR‐432 to activate the Wnt/β‐catenin signaling for modulating the progression of prostate cancer.^12^ Also, lncRNA small nucleolar RNA host gene 3 (SNHG3) has been identified as oncogene in many cancers, such as hepatocellular carcinoma, colorectal cancer, glioma, ovarian cancer, and osteosarcoma.^13^, ^14^, ^15^, ^16^, ^17^ However, the role of SNHG3 is still not clear in prostate cancer. In our study, we scrutinized the role and mechanism of SNHG3 in prostate cancer.

Smad ubiquitination regulatory factor 1 (SMURF1) is HECT‐type E3 ubiquitin ligase and regulates Smad protein stability in the TGF‐β/BMP signaling pathway.^18^ SMURF1 is widely identified as an oncogene in various cancers. SMURF1‐mediated ubiquitination of ARHGAP26 promotes cell migration and invasion in ovarian cancer cells.^19^ SMURF1 facilitates hypopharyngeal carcinoma cell proliferation, migration, and invasion via the mTOR signaling pathway.^20^ Down‐regulation of SMURF1 inhibits ovarian cancer invasion and EMT (Epithelial‐mesenchymal transition) process through modulation on DAB2IP/AKT/Skp2 axis. In this study, the functional role of SMURF1 in prostate cancer was unveiled. The regulatory effects of SNHG3 on SMURF1 were also probed, which might shed a new insight into the molecular mechanism of prostate development.

## 2 MATERIALS AND METHODS

### 2.1 Cell culture

Human prostatic epithelial cell line (RWPE‐1) and prostate cancer cell lines (PC3, DU145, 22RV1 and LNCaP) were obtained from the Cell Bank of the Chinese Academy of Science (Shanghai, China), and maintained with Dulbecco's modified Eagle's medium (DMEM; Gibco, Carlsbad, CA, USA) containing 10% fetal bovine serum (FBS; Gibco) and 1% antibiotics (Gibco). All cells were placed in an incubator at 37°C and 5% CO_2_.

### 2.2 Cell transfection

PC3 or DU145 cells at the confluence of 50%‐80% were collected and transfected with the shRNAs (short hairpin RNAs) specific to SNHG3 (sh‐SNHG3#1 and sh‐SNHG3#2) or sh‐NC (negative control) (Genepharma, SC, CA, USA). Simultaneously, miR‐577 mimics and NC‐mimics were constructed by RiboBio. The pcDNA3.1 targeting SMURF1 and empty pcDNA3.1 vector were purchased from Genechem (Shanghai, China). Transfection was performed with Lipofectamine 3000 (Invitrogen). Relevant sequences were provided in Table S1.

### 2.3 RNA Isolation and qRT‐PCR (quantitative Real‐time polymerase chain reaction)

TRIzol reagent acquired from Invitrogen was initially used for extracting of total RNA. Total RNA was subsequently reverse transcribed to cDNA in standard conditions via a PrimeScript RT Reagent Kit (Takara, Dalian, China). Real‐time PCR analyses were carried out using SYBR Premix Ex Taq (Takara) as the supplier required. Data were all normalized to the expression of GAPDH or U6 and collected on the basis of the 2^−ΔΔCT^ method. PCR primers were provided in Table S1.

### 2.4 CCK‐8 (A cell counting kit‐8) assay

CCK‐8 kit (Dojindo Laboratories, Kumamoto, Japan) was utilized based on standard guides to conduct cell proliferation assay. Transfected PC3 or DU145 cells were placed into 96‐well cell culture plates and then treated by CCK‐8 reagent (10 μL) at 0, 24, 48, 72, and 96 hours. After 2 hours, optical density (OD) was read at 450 nm via a Varioskan Flash Spectral Scanning Multimode Reader (Thermo Fisher Scientific, Waltham, MA, USA).

### 2.5 Colony formation assays

To perform colony formation assays, transfected PC3 or DU145 cells were suspended and planted into six‐well plates. After 14 days, colonies were formed and fixed for 30 minutes by 500 μL of 4% paraformaldehyde (PFA; Solarbio, Beijing, China) and stained for 25 minutes with crystal violet (Sigma‐Aldrich, St. Louis, MO, USA). Colonies were finally counted following photographing.

### 2.6 Transwell assay

For cell migration assay, PC3 or DU145 cells after transfection were starved for 12 hours and subsequently loaded to the Transwell upper chambers. At the same time, DMEM adding 0.5% FBS was added to the lower chambers. The upper chambers were removed following 24 hours, and cells moving into the lower chambers were fixed by 4% PFA and stained with crystal violet. Cell invasion assay was performed similarly using transwell upper chamber coated with Matrigel (BD Biosciences). Results were evaluated via the Leica fluorescent microscope (Leica Microsystems GmbH).

### 2.7 Flow cytometer assay

Cell apoptosis rate was monitored according to the instruction of Annexin‐V fluorescein isothiocyanate (FITC)/propidium iodide (PI) dual staining kit (BD Biosciences). Transfected cells in six‐well plates were prepared for double‐staining for 15 minutes, then subjected to flow cytometry (BD Biosciences).

### 2.8 Subcellular fractionation assay

Extraction of nuclear and cytosolic fractions was conducted via a PARIS Kit purchased from Invitrogen. RNAs in the nuclei and cytoplasm of PC3 or DU145 cells were separated and extracted. Expression levels of SNHG3 in the nuclei and cytoplasm were detected with qRT‐PCR. GAPDH or U6 was examined as fractionation indicators.

### 2.9 Western blot assay

Transfected PC3 or DU145 cells were dissolved by radioimmunoprecipitation Assay lysis solution (Thermo Fisher Scientific). The protein samples were separated using 10% SDS‐PAGE (Sodium dodecyl sulfate‐polyacrylamide gel electrophoresis) (Bio‐Rad Laboratories), transferred into polyvinylidene fluoride (PVDF) membranes (Millipore) afterwards. The membranes were blocked and cultured with primary antibodies, including anti‐E‐cadherin antibody (1/50, ab1416, Abcam, Cambridge, USA), anti‐N‐cadherin antibody (1/1000, ab76057, Abcam), anti‐Bax antibody (1/10000, ab32503, Abcam), anti‐Bcl‐2 antibody (1/2000, ab182858, Abcam), anti‐SMURF1 antibody (1/200, ab38866, Abcam) and anti‐GAPDH antibody (1/2000, ab245357, Abcam). Next day, the membranes were incubated with a secondary antibody after washed thrice. Protein bands were quantified by Odyssey CLx v2.1 software (Media Cybernetics). GAPDH was considered as a loading control.

### 2.10 TUNEL (terminal deoxynucleotidyl transferase dUTP nick end labeling) staining assay

Cell apoptosis was calculated by the Situ Cell Death Detection Kit (Roche) in line with the instructions. After TUNEL staining, cells were dyed by 4,6‐diamino‐2‐phenyl indole (DAPI; Koritai Biotechnology) or Merge (Thermo Fisher Scientific) and observed through a laser scanning confocal microscope (Olympus).

### 2.11 Luciferase reporter assay

Transfection was performed for 48 hours as usual. In brief, SNHG3‐Wt/Mut or SMURF1‐Wt/Mut was co‐transfected into PC3 or DU145 cells with miR‐577 mimics or NC mimics via Lipofectamine 3000. A Dual Luciferase Reporter Assay Kit (Promega) base on the standard instructions was applied afterwards.

### 2.12 RNA immunoprecipitation (RIP) assay

RNA immunoprecipitation (RIP) experiments were performed via the Magna RIP™ RNA‐Binding Protein Immunoprecipitation Kit (Millipore) in line with the protocol offered by its supplier. Antibodies against Ago‐2 and IgG were employed for RIP assay.

### 2.13 RNA pull‐down assay

For the pull‐down assays, PC3 or DU145 cells were transfected with biotinylated SNHG3 (SNHG3 probe‐biotin) or a negative control probe (SNHG3 probe‐no biotin). M‐280 streptavidin magnetic beads (Invitrogen) were utilized to incubate cell lysates after 48 hours of transfections. Finally, qRT‐PCR assay was used to measure the miR‐557 level.

### 2.14 Tumor xenograft model in nude mice

PC3 or DU145 cells transfected with sh‐SNHG3#1 or sh‐NC were subcutaneously inoculated into 6‐week‐old male BALB/c nude mice from the Chinese Academy of Sciences. The tumor volume was tested every 4 days and calculated using the following formula: V = ab^2^/2 (a = tumor length and b = tumor width). Finally, the mice were sacrificed, tumor was excised. Tissues extracted from tumor were fixed in 4% PFA, followed by HE staining. HE staining refers to hematoxylin‐eosin staining and was used to observe tissue structure. Immunohistochemistry (IHC) analysis was then conducted using antibody against Ki67 and SMURF1 (Abcam).

### 2.15 Statistical analysis

Statistical analysis was carried out with GraphPad Prism 7.0 (GraphPad Software) and SPSS Vision 19.0 (SPSS). Differences between groups were analyzed with Student's t test or one‐way ANOVA (analysis of variance). Data from at least three independent experiments were exhibited as mean ± standard deviation (SD). *P* value threshold was set as 0.05 to indicate the statistical significance.

## 3 RESULTS

### 3.1 Inhibited SNHG3 could suppress the progression of prostate cancer cells

**FIGURE 1:**
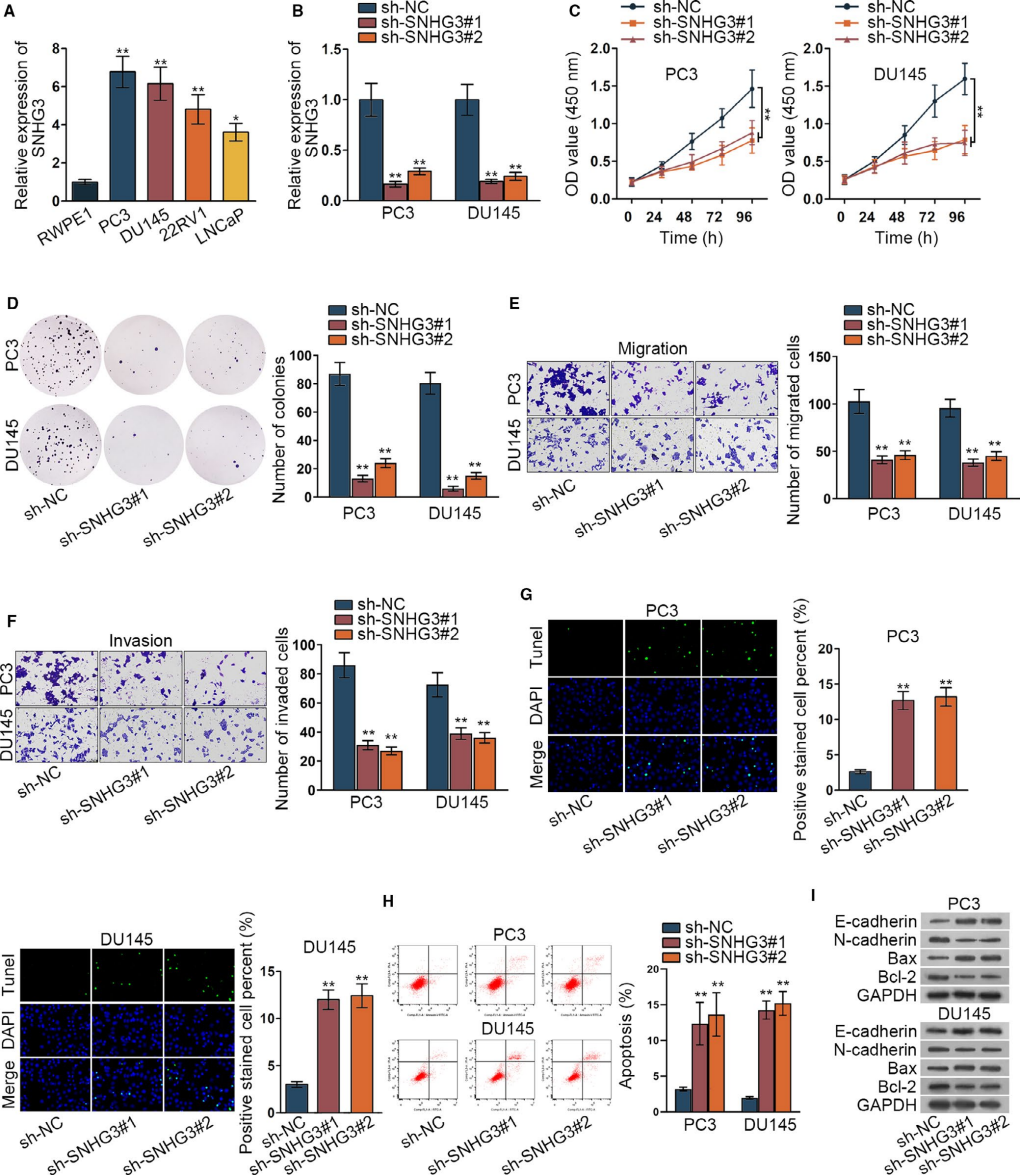
Inhibited SNHG3 could suppress the progression of prostate cancer cells. (A) The expression of SNHG3 was detected in RWPE1 and prostate cancer cells (PC3, DU145, 22RV1, LNCaP) via qRT‐PCR assay. (B) Inhibition efficiency of SNHG3 was assessed in PC3 and DU145 cells via qRT‐PCR assay. (C‐D) CCK‐8 assay and colony formation assay detected the viability and proliferation situation of prostate cancer cells when SNHG3 was inhibited. (E‐F) The transwell assay was implemented to detect the cell migration and invasion when SNHG3 was inhibited. (G‐H) TUNEL assay and flow cytometry analysis investigated the cell apoptosis with silenced SNHG3. (I) The western blot assay was made to test the protein concentration of apoptosis and EMT‐related proteins. *P < .05, **P < .01

LncRNA SNHG3 has been studied as an oncogene in many cancers, such as hepatocellular carcinoma, colorectal cancer, glioma, ovarian cancer, and osteosarcoma.^13^, ^14^, ^15^, ^16^, ^17^ In our study, we assessed the expression of SNHG3 in human normal prostate epithelial cells (RWPE1) and prostate cancer cells (PC3, DU145, 22RV1, LNCaP) at the beginning using qRT‐PCR assay. We found SNHG3 was significantly highly expressed in prostate cancer cells, especially in PC3 and DU145 cells (Figure 1A, *P < .05, **P < .01). Thence, we chose PC3 and DU145 cells for next assays. Also, we inhibited SNHG3 and assessed the inhibition efficiency of SNHG3 in PC3 and DU145 cells (Figure 1B, **P < .01). Then, CCK‐8 assay and colony formation assay detected the cell viability and proliferation. Results indicated that OD value and number of colonies of PC3 and DU145 cells were significantly decreased by SNHG3 depletion (Figure 1C,D, **P < .01). Also, cell migration and invasion were assessed by transwell assay, and we found both migration and invasion were inhibited by silenced SNHG3 (Figure 1E,F, **P < .01). Furthermore, TUNEL assay and flow cytometry analysis investigated the cell apoptosis. Results depicted that the cell apoptosis of PC3 and DU145 were significantly elevated by silenced SNHG3 (Figure 1G,H, **P < .01). In the end, western blot assay was made to test the protein concentration of apoptosis related proteins (Bax and Bcl‐2) and EMT related proteins (E‐cadherin and N‐cadherin). Results showed that protein level of E‐cadherin and Bax were elevated by inhibited SNHG3 and meanwhile protein concentration of N‐cadherin and Bcl‐2 were inhibited by SNHG3 depletion (Figure 1I), which indicated that the cell apoptosis was promoted and EMT progress was inhibited by SNHG3 depletion. Besides, we repeated the function assays with the un‐transfected group (Mock group) as the control. The results depicted that Mock group had no effects on cell biological functions and sh‐SNHG3#1/2 significantly reduced cell viability, proliferation, migration, invasion, EMT process, and elevated cell apoptosis (Figure S1A‐G, **P < .01). In conclusion, inhibited SNHG3 could suppress the progression of prostate cancer cells.

### 3.2 MiR‐577 could bind to SNHG3 and regulate the progression of prostate cancer

**FIGURE 2:**
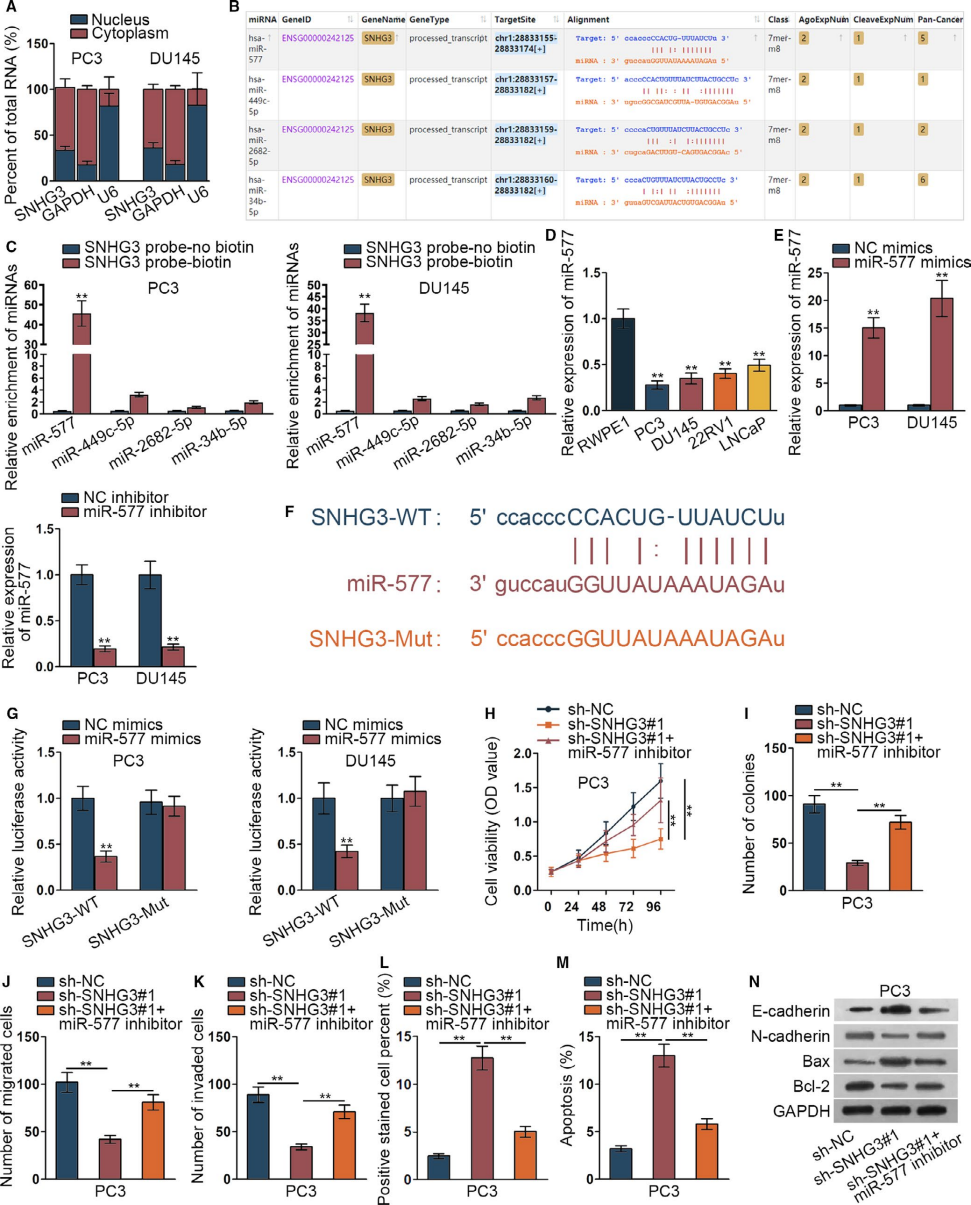
MiR‐577 could bind to SNHG3 and regulate the progression of prostate cancer. (A) The subcellular fraction assay revealed subcellular location of SNHG3 in PC3 and DU145 cells. (B) ENCORI (http://starbase.sysu.edu.cn/index.php) was implemented to detect potential miRNAs. (C) RNA pull down assay verified the binding among four miRNAs and SNHG3. (D) The miR‐577 expression in RWPE1 and prostate cancer cells (PC3, DU145, 22RV1, LNCaP) was assessed via qRT‐PCR assay. (E) Inhibition and overexpression efficiency of miR‐577 were investigated by qRT‐PCR assay. (F) The binding site of miR‐577 and SNHG3 was presented according to ENCORI. (G) The luciferase reporter assay was implemented to detect the binding of miR‐577 and SNHG3. (H‐N) Rescue assay of CCK‐8 assay, colony formation assay, tranwell assay, TUNEL assay, flow cytometry analysis, and western blot assay were conducted to know about the relationship of miR‐577 and SNHG3 in PC3 cells. **P < .01

Previous studies have found that lncRNA SNHG3 can sponge to miRNA and regulate the expression of mRNA.^21^ Therefore, we identified the subcellular location of SNHG3 in PC3 and DU145 cells via subcellular fraction assay. Results indicated that SNHG3 mainly located in cytoplasm of PC3 and DU145 cells (Figure 2A). Then ENCORI website was implemented to search suitable miRNAs binding SNHG3. Four miRNAs were searched out by CLIP data: medium stringency (≥2) and degradome data: low stringency (≥1) (Figure 2B). Furthermore, RNA pull down assay verified the binding of 4 miRNAs (miR‐577, miR‐449c‐5p, miR‐2682‐5p and miR‐34b‐5p) to SNHG3. We found that only miR‐577 was significantly pulled down by biotin‐labeled SNHG3 probe while no products were pulled down by no biotin‐labeled SNHG3 probe (Figure 2C, **P < .01). Thence, we detected the expression of miR‐577 in prostate cancer cells via qRT‐PCR assay. MiR‐577 was significantly down‐regulated in prostate cancer cells (Figure 2D, **P < .01). Also, inhibition and overexpression efficiency of miR‐577 was investigated by qRT‐PCR assay (Figure 2E, **P < .01). We presented the binding site of miR‐577 and SNHG3 (Figure 2F). For verifying the binding of miR‐577 and SNHG3, luciferase reporter assay was implemented. We found that overexpressed miR‐577 inhibited the relative luciferase activity of SNHG3 wild type, which proved the binding of miR‐577 and SNHG3 (Figure 2G, **P < .01). Finally, we conducted recue assay to verify the relationship of miR‐577 and SNHG3 in PC3 cells. The results demonstrated that decreased cell viability and proliferation via inhibited SNHG3 was recovered by miR‐577 depletion (Figure 2H‐I, **P < .01). Also, decreased cell migration and invasion via SNHG3 silence was offset by miR‐577 depletion (Figure 2J‐K, **P < .01). Elevated cell apoptosis and hindered EMT process by SNHG3 silence was countervailed by miR‐577 inhibition (Figure 2L‐N, **P < .01). In conclusion, miR‐577 could bind to SNHG3 and regulate the progression of prostate cancer.

### 3.3 SNHG3/miR‐577/SMURF1 axis was found in prostate cancer cells

**FIGURE 3:**
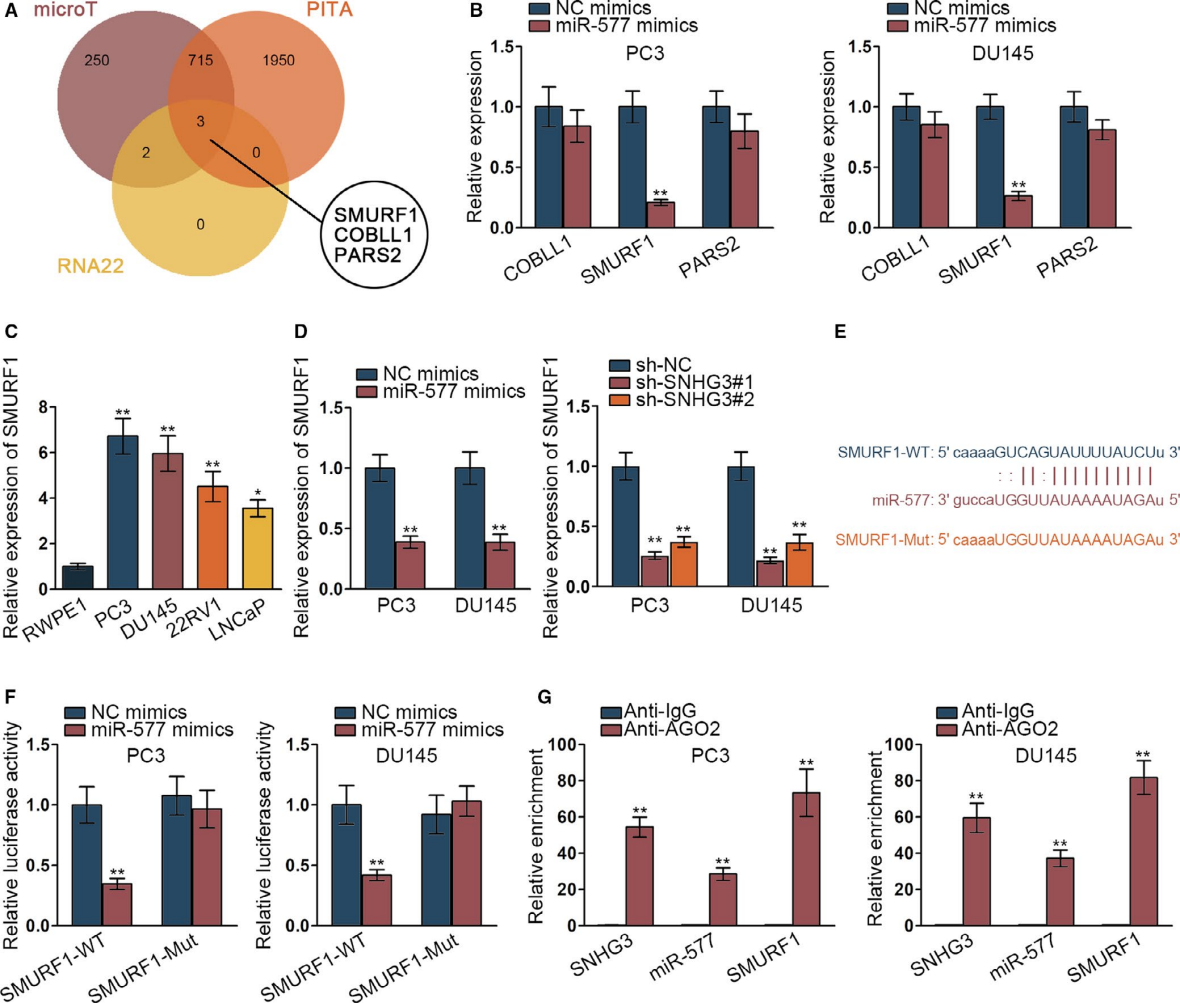
SNHG3/miR‐577/SMURF1 axis was found in prostate cancer cells. (A) The potential mRNAs were searched via ENCORI website. (B) The qRT‐PCR assay was implemented to detect expression of three mRNAs under miR‐577 mimics. (C) SMURF1 expression was detected in prostate cancer cells via qRT‐PCR assay. (D) The expression of SMURF1 was tested when miR‐577 was overexpressed or SNHG3 was silenced by qRT‐PCR. (E) The binding site of SMURF1 and miR‐577 was presented via ENCORI website. (F‐G) Luciferase reporter assay and RIP assay verified the binding of SMURF1 and miR‐577. *P < .05, **P < .01

We further detected the target gene of miR‐577 and used ENCORI website to search potential mRNAs binding miR‐577. SMURF1, COBLL1 and PARS2 were simultaneously screened by microT, PITA, RNA22 prediction (Figure 3A). Thence, qRT‐PCR assay was implemented to detect the expression of the 3 mRNAs under miR‐577 mimics. Results showed that SMURF1 expression was significantly decreased by miR‐577 overexpression (Figure 3B, **P < .01). Therefore, SMURF1 expression was detected in prostate cancer cells via qRT‐PCR assay. SMURF1 was aberrantly highly expressed in prostate cancer cells (Figure 3C, *P < .05, **P < .01). Then we tested the expression of SMURF1 when miR‐577 was overexpressed or SNHG3 was silenced. We discovered that the expression of SMURF1 was remarkably declined in the situation of overexpressing miR‐577 or silencing SNHG3 (Figure 3D, **P < .01). Binding site of SMURF1 and miR‐577 was presented (Figure 3E). We further verified the binding of SMURF1 and miR‐577 via luciferase reporter assay and RIP assay. We found that relative luciferase activity of SMURF1 wild type was significantly inhibited by overexpressed miR‐577 while that of mutant SMURF1 was not impacted (Figure 3F, **P < .01). SNHG3, SMURF1, and miR‐577 were significantly enriched in Ago2‐precitated RISC (RNA induced silence complex) in RIP assay (Figure 3G, **P < .01). In conclusion, SNHG3/miR‐577/SMURF1 axis was found in prostate cancer cells.

### 3.4 SNHG3/miR‐577/SMURF1 axis could modulate the progression of prostate cancer cells

**FIGURE 4:**
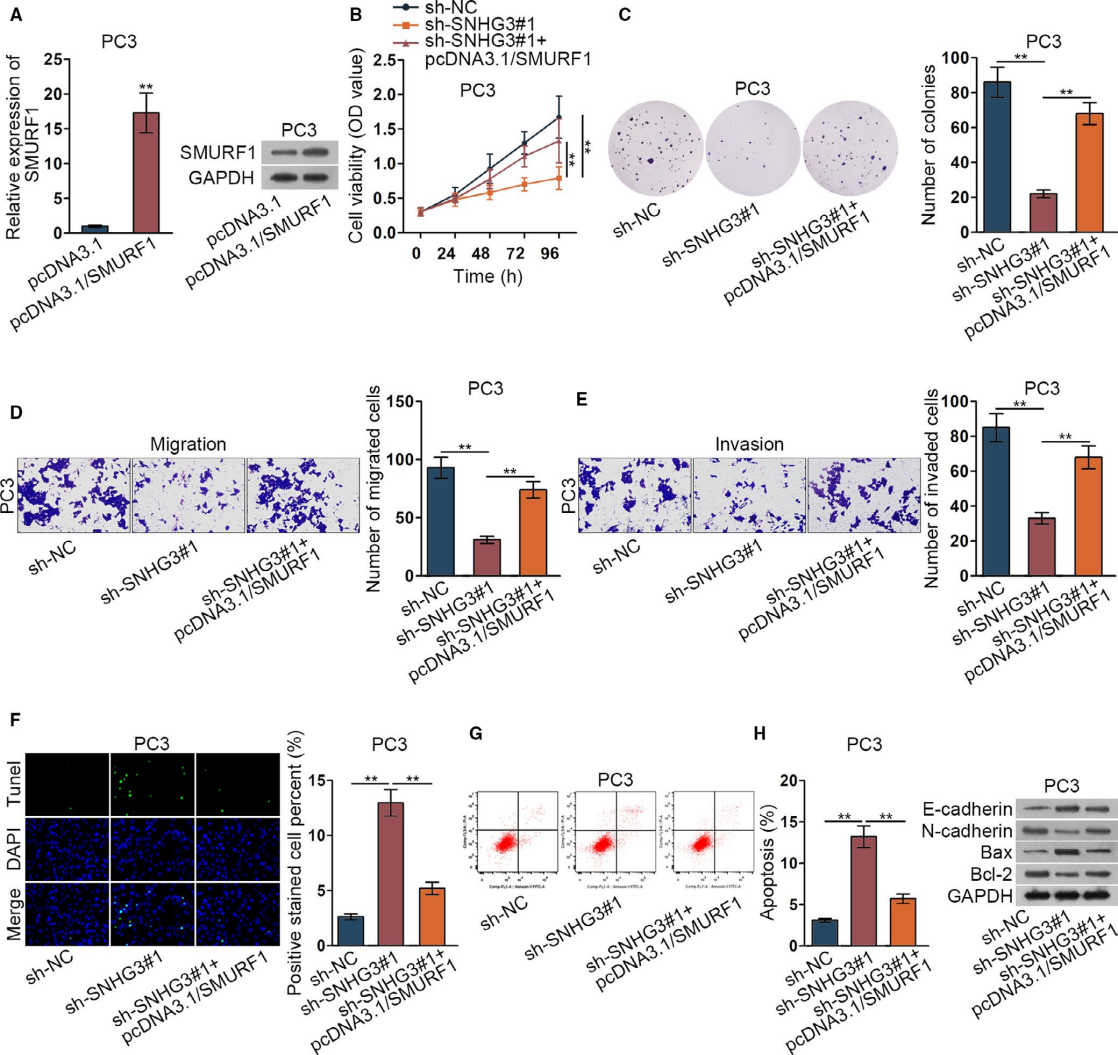
SNHG3/miR‐577/SMURF1 axis could modulate the progression of prostate cancer cells. (A) The overexpression efficiency of SMURF1 was investigated via qRT‐PCR assay in PC3 cells and western blot assay. (B‐H) CCK‐8 assay, colony formation assay, tranwell assay, TUNEL assay, flow cytometry analysis, and western blot assay were implemented to detect the relationship between SNHG3 and SMURF1. **P < .01

We further detected the relationship between SNHG3 and SMURF1. We firstly implemented qRT‐PCR assay and western blot assay to verify the overexpression efficiency of SMURF1 (Figure 4A, **P < .01). Then, CCK‐8 assay and colony formation assay verified that SMURF1 gain of function could recover the inhibitory effect of silenced SNHG3 on PC3 cells viability and proliferation (Figure 4B,C, **P < .01). Transwell assay revealed that SMURF1 gain of function could offset the suppressed cell migration and invasion by silenced SNHG3 (Figure 4D,E, **P < .01). Meanwhile, TUNEL assay and flow cytometry analysis uncovered that elevated cell apoptosis by SNHG3 depletion was recovered by overexpressed SMURF1 (Figure 4F,G, **P < .01). Inhibited EMT progress by inhibited SNHG3 was offset by overexpressed SMURF1 (Figure 4H, **P < .01). In conclusion, SNHG3/miR‐577/SMURF1 axis could modulate the progression of prostate cancer cells.

### 3.5 SNHG3 inhibition could reduce the progression of prostate tumor

**FIGURE 5:**
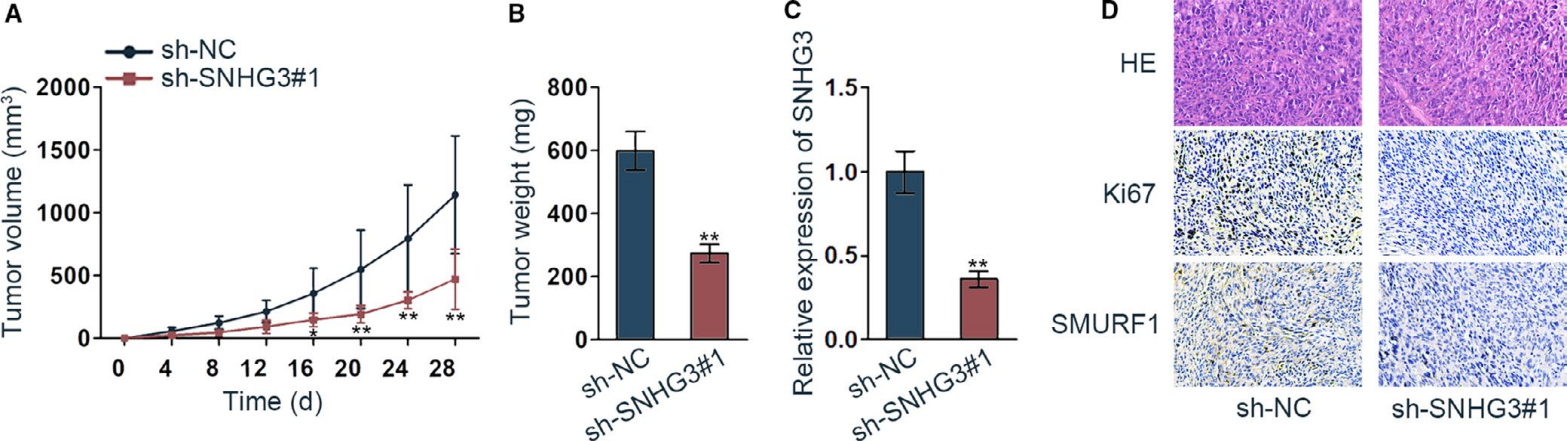
SNHG3 inhibition could reduce the progression of prostate tumor. (A‐B) Tumor size and weight were measured with the change of time. (C) SNHG3 expression was detected in tumor via qRT‐PCR assay. (D) HE staining of mice tissues was applied to observe tissue structure and immunohistochemical staining detected the positive expression of Ki67 and SMURF1 by depletion of SNHG3. **P < .01

We detected the function of SNHG3 in vivo. We found that not only the tumor volume but also the tumor weight was decreased with inhibited SNHG3 (Figure 5A,B, *P < .05, **P < .01). Also, qRT‐PCR assay revealed that SNHG3 expression in mice tissue was significantly decreased by silenced SNHG3 (Figure 5C, **P < .01). Meanwhile, HE staining of mice tissues by silenced SNHG3 was exhibited; immunohistochemical method depicted that Ki67 and SMURF1 positivity were both decreased by silenced SNHG3 (Figure 5D). In conclusion, SNHG3 inhibition could reduce the progression of prostate tumor.

## 4 DISCUSSION

LncRNAs have been identified that they can modulate the expression of miRNAs and mRNAs in cancer and regulate the progression of cancer.^22^, ^23^, ^24^, ^25^ Previous studies have demonstrated the role of lncRNA SNHG3 in cancers. For instance, up‐regulated lncRNA SNHG3 is connected to the serious progression and poor prognosis of hepatocellular carcinoma.^13^ SNHG3 can promote the progression of glioma by reducing the expression of KLF2 and p21.^15^ Also, lncRNA SNHG3 can modulate GSK3β/β‐catenin pathway to regulate the progression of ovarian cancer.^16^ In our study, we firstly detected the expression of SNHG3 in prostate cancer cells using qRT‐PCR assay. We found that SNHG3 was aberrantly up‐regulated in prostate cancer cells than in human normal prostate epithelial cells. Next, the oncogenic role of SNHG3 in prostate cancer was verified through in‐vitro and in‐vivo assays.

It was widely reported that lncRNAs serve as competing endogenous RNAs (ceRNAs) through competing with mRNAs to bind with miRNAs. The ceRNA is a typical regulatory pattern at post‐transcription level in cancers and SNHG3 was widely involved in the ceRNA pattern. SNHG3 can function as a ceRNA by acting as miR‐182‐5p sponge to indirectly regulate expression of mRNAs and aggravate the progression of colorectal cancer.^14^ SNHG3 forms an SNHG3/miRNA‐151a‐3p/RAB22A pathway to regulate the cell migration in osteosarcoma.^17^ SNHG3 activates the progression of clear cell renal cell carcinoma via regulating the expression of miR‐139‐5p and as a result to up‐regulate TOP2A expression.^26^ SNHG3 can modulate the cell growth and migration of laryngeal carcinoma by modulating miR‐384/WEE1 pathway.^27^ In our study, we detected the subcellular location of SNHG3 and the results revealed that SNHG3 was mainly located on the cytoplasm of prostate cancer cells. We then sought to search for the ceRNA axis with the involvement of SNHG3 in prostate cancer cells.

Based on bio‐informatics analysis and mechanism assays, SNHG3 could bind with miR‐577, and inhibited miR‐577 could rescue the effects of inhibited SNHG3 on cell biological functions. MiR‐577 was reported to repress cell proliferation and EMT process via suppression on Wnt/β‐catenin pathway in non‐small cell lung cancer.^28^ MiR‐577 is sponged by ZEB1‐AS1 and reverses the tumor‐facilitating effects of ZEB1‐AS1 on glioma cells.^29^ MiR‐577 was also reported in the ceRNA axis in various cancers. A positive TMPO‐AS1/miR‐577/RAB14 feedback loop facilitates the progression of cervical cancer.^30^ A positive DUXAP8/miR‐577/RAB14 feedback loop promotes cell migration and invasion in colorectal cancer.^31^ Next, we identified SMURF1 as the downstream target of miR‐577. SMURF1 is HECT‐type E3 ubiquitin ligase and functions as tumor facilitator in diverse cancers.^19^, ^20^ SMURF1 was negatively modulated by miR‐577 and was positively regulated by SNHG3. SNHG3 served as the ceRNA against miR‐577 to up‐regulate SMURF1. The further rescue assays depicted that SMURF1 completely restore the effects of silenced SNHG3 on cell viability, proliferation, migration, invasion, EMT process and apoptosis. On the whole, SNHG3 endogenously sponged miR‐577 to elevate SMURF1 expression, thus facilitating malignant phenotype of prostate cancer. It is for the first time that the ceRNA axis of SNHG3/miR‐577/SMURF1 was uncovered in prostate cancer cells. We innovatively uncovered the oncogenic role of SNHG3 and SMURF1 in prostate cancer, which might provide a new insight into identifying the biomarkers for prostate cancer.

## CONFLICTS OF INTERESTS

Authors state no conflicts of interest in this study.

## AUTHOR CONTRIBUTION

Teng Li and Yi Xing: contributed to the conception of the study. Fan Yang and Yangyang Sun contributed significantly to analysis and manuscript preparation. Shaojin Zhang and Qingwei Wang: performed the data analyses and wrote the manuscript. Weixing Zhang: helped perform the analysis with constructive discussions.

## Supporting information

Fig S1

Table S1

## Data Availability

The data are confidential and cannot be shared.
