# Supplementary figures and images for: LncRNA SNHG3 sponges miR‐577 to up‐regulate SMURF1 expression in prostate cancer
Source: Cancer Med. 2020 Apr 5;9(11):3852–62. doi: 10.1002/cam4.2992 (PMC7286463; doi:10.1002/cam4.2992)

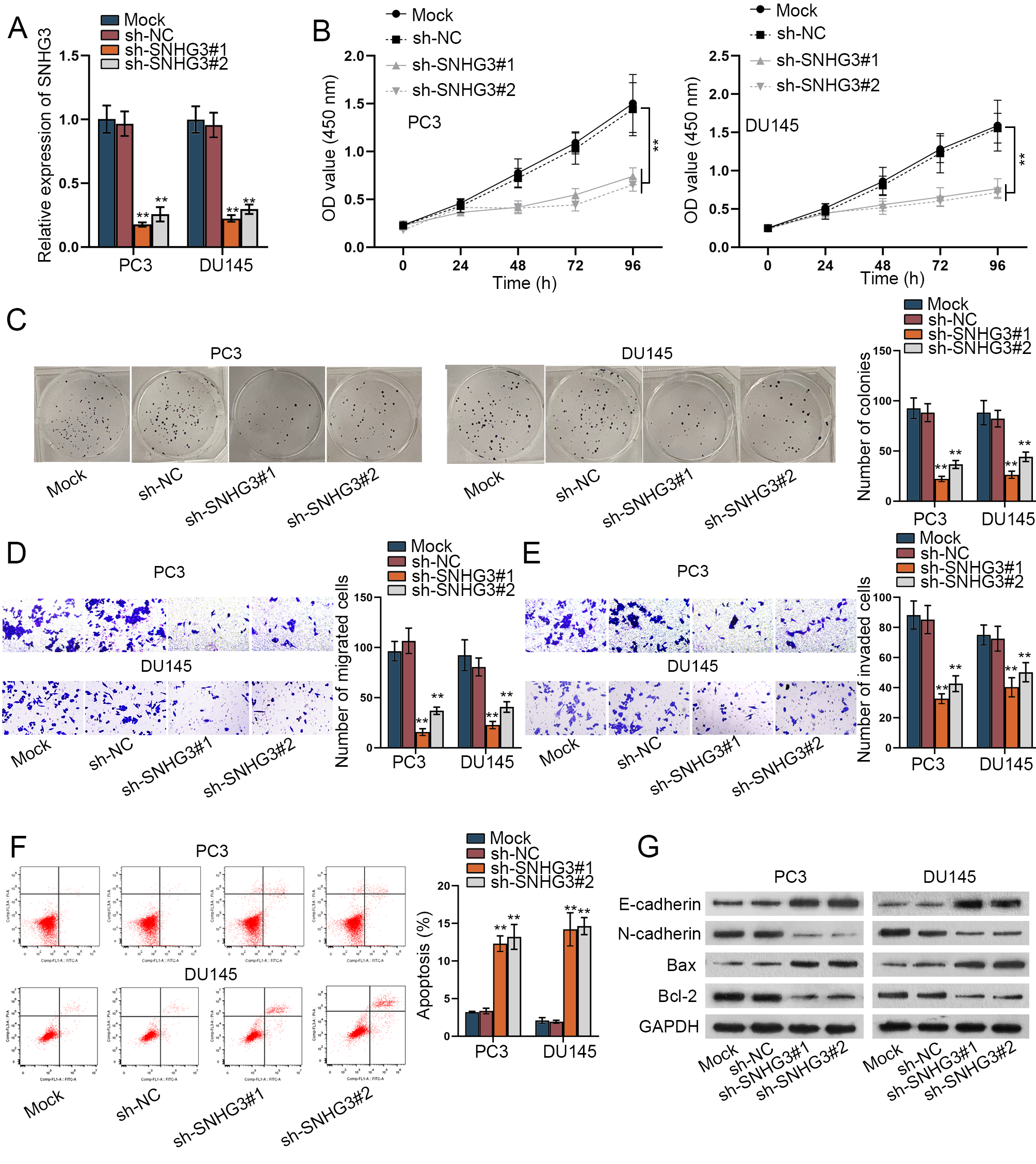

Supplement: Supplementary file 1 — Fig S1 [file CAM4-9-3852-s001.tif]
